# Combined effects of progesterone and *SOCS3* DNA methylation on T2DM: a case–control study

**DOI:** 10.1186/s13148-021-01172-9

**Published:** 2021-09-26

**Authors:** Lulu Wang, Zhenxing Mao, Xiaotian Liu, Dandan Wei, Pengling Liu, Luting Nie, Keliang Fan, Ning Kang, Yu Song, Qingqing Xu, Juan Wang, Mian Wang, Wei Liao, Tao Jing, Wenjie Li, Chongjian Wang, Wenqian Huo

**Affiliations:** 1 Department of Epidemiology and Biostatistics, College of Public Health, Zhengzhou University, Zhengzhou, Henan People’s Republic of China; 2 Department of Occupational and Environmental Health Sciences, College of Public Health, Zhengzhou University, 100 Kexue Avenue, Zhengzhou, 450001 Henan People’s Republic of China; 3 Department of Nutrition and Food Hygiene, College of Public Health, Zhengzhou University, Zhengzhou, Henan People’s Republic of China; 4 School of Public Health, Tongji Medical College, Huazhong University of Science and Technology, Wuhan, Hubei People’s Republic of China

**Keywords:** Type 2 diabetes mellitus, Progesterone, Suppressor of cytokine signaling 3, Methylation, Combined effect

## Abstract

**Background:**

This study aims to investigate the independent and combined effects of progesterone and *suppressor of cytokine signaling* (*SOCS*)-3 DNA methylation on type 2 diabetes mellitus (T2DM) among men and postmenopausal women in rural China.

**Methods:**

A case–control study with 914 participants (329 T2DM, 585 controls) was conducted. Serum progesterone was detected with liquid chromatography-tandem mass spectrometry. DNA methylation of *SOCS3* was determined by MethylTarget™. Linear regression was applied to evaluate the associations of progesterone and *SOCS3* methylation with marks of glucose metabolism. Logistic regression was employed to investigate the independent and combined effects of progesterone and *SOCS3* methylation with T2DM in men and postmenopausal women.

**Results:**

After multiple adjustment, progesterone was positively associated with T2DM in both men (odds ratio (OR) (95% confidence interval (CI)): 2.77 (1.79, 4.29)) and postmenopausal women (OR (95% CI): 1.85 (1.26, 2.72)). Methylation level of *Chr17:76,356,190* or *Chr17:76,356,199* (*SOCS3*) was negatively associated with T2DM in both men (OR (95% CI): 0.58 (0.39, 0.86) or 0.27 (0.14, 0.51)) and postmenopausal women (OR (95% CI): 0.43 (0.29, 0.65) or 0.53 (0.28, 0.99)). Subjects with high progesterone and low *Chr17:76,356,190* or *Chr17:76,356,199* methylation were more susceptible to have a higher prevalence of T2DM (men: OR (95% CI): 5.20 (2.49, 10.85) or 5.62 (2.74, 11.54); postmenopausal women: OR (95% CI): 3.66 (1.85, 7.26) or 3.27 (1.66, 6.45)).

**Conclusions:**

The independent and combined effects of progesterone and *SOCS3* methylation on T2DM were found among men and postmenopausal women, suggesting that ensuring low levels of progesterone and high methylation of *SOCS3* could reduce the prevalence of T2DM.

**Trial registration** The Chinese Clinical Trial registration: The Henan Rural Cohort Study, ChiCTR-OOC-15006699. Registered 06 July 2015, http://www.chictr.org.cn/showproj.aspx?proj=11375

**Supplementary Information:**

The online version contains supplementary material available at 10.1186/s13148-021-01172-9.

## Background

Diabetes is a common chronic metabolic disease with high prevalence and disease burden. According to the International Diabetes Federation (IDF) Diabetes Atlas, 1 in 11 adults aged 20–79 years have diabetes nowadays, which means that the number of adults with diabetes has reached 463 million, and is estimated to reach 578 million by 2030, and 700 million by 2045 [1]. Furthermore, China has the largest number of diabetes patients, and the majority are type 2 diabetes mellitus (T2DM) [1]. T2DM and its complications place a significant financial burden on individuals, families, and healthcare systems, especially in rural areas, in which income levels and education attainment are low [2]. Therefore, it is urgent to find out and control the related factors that lead to hyperglycemia and T2DM.

In recent years, a growing number of researches have reported that steroid hormones may influence the incidence of diabetes [3]. Progesterone, a kind of steroid hormones, plays an integral role in many physiological systems, including reproduction [4] and neuroprotection [5]. However, an excess of progesterone has been implicated in the pathogenesis of metabolic disorders [6], such as diabetes, by affecting pancreatic β-cell apoptosis and then causing impairment of insulin production and secretion [7], or causing insulin resistance (IR) [8]. Experiments on animals have demonstrated that mice knockout progesterone receptor (PR) has higher levels of insulin and lower levels of fasting glucose than the controls [9]. Furthermore, population-based studies have reported that pregnancy and use of progestin-only contraception are linked with the risk of developing diabetes in Hispanic women [10]. A recent cross-sectional study drawn from the KORA F4/FF4 cohort study which were followed up for approximately 6.4 years, reported an inverse association of progesterone with fasting insulin, and a positive association of progesterone with quantitative insulin sensitivity check index (QUICKI) among men. Additionally, our previous study found that high levels of progesterone were correlated with a higher chance of presenting T2DM among men and postmenopausal women [11]. Nevertheless, a cross-sectional study reported a decrease in progesterone levels among T2DM patients compared with those in control group [12]. Taken together, there are few population studies on the relationship between progesterone and T2DM, and the results are inconsistent. Importantly, previous evidence from pregnant population is much limited [13, 14], so it is essential to explore the correlations among general population.

There was evidence that epigenetic changes were important in the development of T2DM [15, 16]. SOCS family proteins form part of a classical negative feedback system that regulates cytokine signal transduction, which includes SOCS1-7 as well as cytokine-inducible SH2-containing protein (CIS). Among this protein family, SOCS3 has received special attention in recent years, and proposed that it could cause IR by interacting with insulin receptors, inducing signaling protein degradation, or participating in the signaling of other cytokines [17], thereby reduces the uptake and clearance of glucose by insulin in the surrounding tissues. Additionally, some studies found that SOCS3 could inhibit phosphatidylinositol 3-kinase (PI3K)/Akt phosphorylation in insulin signaling pathway to reduce hepatic insulin sensitivity [18, 19]. Meanwhile, progesterone could also suppress the PI3K/Akt pathway and then lead to IR [20]. Moreover, there was animal trail reported that SOCS3 mRNA level that measured within the arcuate nucleus was significantly reduced in the rats who were ovariectomized on day 18 of pregnancy and treated with delayed progesterone withdrawal, compared to those treated with normal progesterone withdrawal [21]. In addition, our previous study found a negative association of *Chr17:76356190* and *Chr17:76356199* (*SOCS3*) methylation with T2DM [22]. Therefore, it is reasonable to assume that progesterone and *SOCS3* methylation might have the combined effects on T2DM.

Thus, using the data from the Henan Rural cohort, the present research aimed to (1) assess the associations of progesterone and methylation of *Chr17:76356190* and *Chr17:76356199* (*SOCS3*) with T2DM; and (2) further examine the combined effects of progesterone and *SOCS3* methylation on T2DM.

## Results

### Basic characteristics

The basic characteristics of participants are summarized in Table 1. Compared with control group, individuals with T2DM were more likely to have higher body mass index (BMI), pulse pressure (PP), triglyceride (TG), fasting plasma glucose (FPG), insulin (INS), glycosylated hemoglobin A1c (HbA1c), progesterone levels and lower levels of *Chr17:76356190* and *Chr17:76356199* methylation, as well as had family history of T2DM (all *P* < 0.05) in both men and postmenopausal women. Furthermore, T2DM patients tended to be smokers and drinkers in men, and tended to have higher levels of total cholesterol (TC) in postmenopausal women (all *P* < 0.05).

**Table 1:** Basic characteristics of the study population among men and postmenopausal women

Variables	Men			Postmenopausal women		
	Control	T2DM	*P*	Control	T2DM	*P*
Subjects, n	286	157		299	172	
Age (years)	61.00 (54.00, 65.00)	61.00 (54.00, 65.00)	0.653	63.00 (58.00, 66.00)	63.00 (58.00, 66.00)	0.845
*Marital status, n (%)*			0.912			0.354
Married/cohabiting	256 (89.5)	140 (89.2)		259 (86.6)	154 (89.5)	
Widowed/single/divorced/separation	30 (10.5)	17 (10.8)		40 (13.4)	18 (10.5)	
*Smoking status, n (%)*			0.019			1.000
Never smoking	102 (35.7)	53 (33.8)		298 (99.7)	172 (100.0)	
Give up smoking	44 (15.4)	41 (26.1)		0	0	
Smoking now	140 (49.0)	63 (40.1)		1 (0.3)	0	
*Drinking status, n (%)*			0.005			1.000
Never drinking	170 (59.4)	70 (44.6)		292 (97.7)	168 (97.7)	
Stop drinking	38 (13.3)	22 (14.0)		0	0	
Drinking now	78 (27.3)	65 (41.4)		7 (2.3)	4 (2.3)	
More vegetable and fruit intake, n (%)	186 (65.0)	104 (66.2)	0.798	190 (63.5)	105 (61.0)	0.589
*Physical activity, n (%)*			0.664			0.121
Low	83 (29.0)	52 (33.1)		64 (21.4)	50 (29.1)	
Mediate	101 (35.3)	53 (33.8)		161 (53.8)	89 (51.7)	
High	102 (35.7)	52 (33.1)		74 (24.7)	33 (19.2)	
*Level of education, n (%)*			0.189			0.648
≤ Primary school	124 (43.4)	58 (36.9)		206 (68.9)	115 (66.9)	
Junior secondary and above	162 (56.6)	99 (63.1)		93 (31.1)	57 (33.1)	
Family history of T2DM, n (%)	5 (1.7)	12 (7.6)	0.002	1 (0.3)	10 (5.8)	< 0.001
BMI (kg/m^2^)	22.89 (20.94, 25.14)	25.83 (23.51, 29.16)	< 0.001	23.08 (21.30, 25.45)	27.19 (23.83, 29.09)	< 0.001
PP (mmHg)	44.50 (40.00, 51.00)	47.00 (42.00, 55.00)	0.025	50.00 (42.00, 57.00)	54.00 (46.00, 62.00)	0.001
TC (mmol/L)	4.44 (3.93, 4.94)	4.53 (4.00, 5.30)	0.184	4.85 (0.82)	5.05 (1.02)	0.028
TG (mmol/L)	1.39 (0.96, 2.16)	1.71 (1.19, 2.89)	< 0.001	1.54 (1.13, 2.34)	1.97 (1.42, 2.91)	< 0.001
FPG (mmol/L)	5.01 (4.69, 5.40)	8.25 (7.27, 10.34)	< 0.001	5.17 (0.58)	8.10 (7.29, 10.12)	< 0.001
INS (pmol/L)	82.09 (64.03, 106.19)	92.65 (69.24, 125.77)	0.001	85.42 (67.85, 108.52)	108.45 (83.51, 147.30)	< 0.001
HbA1c (%)	5.50 (5.30, 5.80)	7.50 (6.60, 9.10)	< 0.001	5.60 (5.30, 5.80)	7.60 (6.70, 9.05)	< 0.001
Methylation level of SOCS3 (Chr17:76356190) (%), mean ± SD	0.99 (0.67, 1.60)	0.79 (0.61, 1.03)	< 0.001	0.92 (0.63, 1.55)	0.77 (0.54, 1.05)	< 0.001
Methylation level of SOCS3 (Chr17:76356199) (%), mean ± SD	0.98 (0.72, 1.31)	0.82 (0.58, 1.03)	< 0.001	0.90(0.69, 1.17)	0.82 (0.66, 1.04)	0.014
Progesterone (ng/ml)	0.83 (0.56, 1.16)	1.29 (0.82, 1.77)	< 0.001	0.86 (0.62, 1.17)	1.16 (0.92, 1.64)	< 0.001

Values are presented as mean (standard deviation) or median (interquartile range) for continuous variables and number (percentage) for categorical variables

P values calculated using *t* tests or Mann–Whitney *U* tests and Chi-square tests

BMI, body mass index; FPG, fasting plasma glucose; HbA1c, glycosylated hemoglobin A1c; INS, insulin; PP, pulse pressure; SD, standard deviations; SOCS3, suppressor of cytokine signaling 3; TC, total cholesterol; TG, triglyceride; T2DM, type 2 diabetes mellitus

*P < 0.05 compared with control

### The independent associations of progesterone and methylation levels of *Chr17:76356190* and *Chr17:76356199* with T2DM

Table 2 shows the relationships of progesterone and methylation levels of *Chr17:76356190* and *Chr17:76356199* with T2DM. Overall, there was a strongly positive association between progesterone levels and T2DM before and after adjusting for confounds in men (OR (95% CI): 3.37 (2.34, 4.87) and 2.77 (1.79, 4.29), respectively) and postmenopausal women (OR (95% CI): 2.40 (1.67, 3.44) and 1.85 (1.26, 2.72), respectively). Furthermore, the prevalence of T2DM was increased with the levels of progesterone increasing (*P* for trend < 0.05).

**Table 2:** Associations of progesterone and methylation level of SOCS3 (Chr17:76356190 and Chr17:76356199) with T2DM

Variables	ORs (95% CIs)		
	Model 1	Model 2	Model 3
*Progesterone*			
Men			
Continuous	3.37 (2.34, 4.87)*	3.23 (2.18, 4.77)*	2.77 (1.79, 4.29)*
T1 (≤ 0.76)	Reference	Reference	Reference
T2 (0.76–1.21)	1.56 (0.91, 2.67)	1.44 (0.83, 2.51)	1.50 (0.82, 2.72)
T3 (> 1.21)	5.39 (3.21, 9.05)*	5.08 (2.94, 8.78)*	3.88 (2.11, 7.15)*
*P*-trend	< 0.001	< 0.001	< 0.001
Postmenopausal women			
Continuous	2.40 (1.67, 3.44)*	2.38 (1.65, 3.43)*	1.85 (1.26, 2.72)*
T1 (≤ 0.78)	Reference	Reference	Reference
T2 (0.78 ~ 1.17)	2.25 (1.35, 3.76)*	2.33 (1.38, 3.93)*	1.85 (1.03, 3.34)*
T3 (> 1.17)	4.80 (2.90, 7.94)*	4.78 (2.85, 8.01)*	3.24 (1.79, 5.85)*
*P*-trend	< 0.001	< 0.001	< 0.001
*Methylation of SOCS3 (Chr17:76356,190)*			
Men			
Continuous	0.58 (0.41, 0.82)*	0.62 (0.43, 0.89)*	0.58 (0.39, 0.86)*
T1 (≤ 0.76)	Reference	Reference	Reference
T2 (0.76–1.08)	1.01 (0.64, 1.60)	1.06 (0.66, 1.72)	1.05 (0.62, 1.78)
T3 (> 1.08)	0.39 (0.23, 0.64)*	0.41 (0.25, 0.70)*	0.39 (0.22, 0.70)*
*P*-trend	< 0.001	0.001	0.002
Postmenopausal women			
Continuous	0.43 (0.30, 0.63)*	0.42 (0.28, 0.61)*	0.43 (0.29, 0.65)*
T1 (≤ 0.75)	Reference	Reference	Reference
T2 (0.75–1.04)	1.00 (0.64, 1.56)	0.98 (0.62, 1.55)	1.17 (0.69, 1.98)
T3 (> 1.04)	0.46 (0.28, 0.74)*	0.45 (0.27, 0.74)*	0.51 (0.29, 0.89)*
*P*-trend	0.002	0.002	0.023
*Methylation of SOCS3 (Chr17:76356199)*			
Men			
Continuous	0.31 (0.18, 0.54)*	0.32 (0.18, 0.57)*	0.27 (0.14, 0.51)*
T1 (≤ 0.76)	Reference	Reference	Reference
T2 (0.76–1.08)	0.62 (0.39, 0.99)*	0.55 (0.34, 0.90)*	0.49 (0.28, 0.84)*
T3 (> 1.08)	0.43 (0.26, 0.69)*	0.42 (0.25, 0.70)*	0.39 (0.22, 0.68)*
*P*-trend	0.001	0.001	0.001
Postmenopausal women			
Continuous	0.46 (0.27, 0.79)*	0.45 (0.26, 0.80)*	0.53 (0.28, 0.99)*
T1 (≤ 0.75)	Reference	Reference	Reference
T2 (0.75–1.04)	0.98 (0.63, 1.54)	1.03 (0.65, 1.62)	1.09 (0.65, 1.84)
T3 (> 1.04)	0.54 (0.33, 0.86)*	0.51 (0.31, 0.82)*	0.60 (0.34, 1.04)
*P*-trend	0.011	0.008	0.081

Model 1: no adjust

Model 2: adjusted for age, smoking status, drinking status, physical activity, and family history of T2DM

Model 3: Model 2 + BMI, PP, TC, and TG

CIs, confidence intervals; ORs, odds ratios; SOCS3, suppressor of cytokine signaling 3; T2DM, type 2 diabetes mellitus; T: tertiles

*P < 0.05

After adjusting multiple confounds, *Chr17:76356190* methylation was inversely associated with T2DM in men (OR (95% CI): 0.58 (0.39, 0.86)), and the relationship was slightly stronger in postmenopausal women (OR (95% CI): 0.43 (0.29, 0.65)). Similarly, the inverse relationship between *Chr17:76356199* methylation and T2DM was found both in men (OR (95% CI): 0.27 (0.14, 0.51)) and postmenopausal women (OR (95% CI): 0.53 (0.28, 0.99)). In addition, the risk of T2DM was decreased with the levels of *Chr17:76356190* and *Chr17:76356199* methylation increasing (both *P* for trend < 0.001).

### Correlations of progesterone and methylation levels of *Chr17:76356190* and *Chr17:76356199* with markers of glucose metabolism

**Fig. 1:**
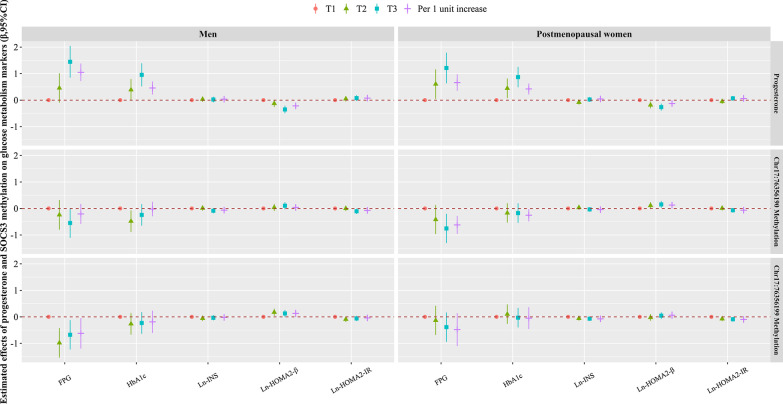
The β coefficients (95% CIs) in markers of glucose metabolism associated with serum progesterone concentrations and methylation levels of SOCS3 (Chr17:76356190 and Chr17:76356199) in men and postmenopausal women. Adjusted for age, smoking status, drinking status, physical activity, family history of T2DM, BMI, PP, TC, and TG. CI, confidence interval; FPG, fasting plasma glucose; HbA1c, glycosylated hemoglobin A1c; Ln-: natural log; HOMA: homeostasis model assessment; INS, insulin; IR: insulin resistance; SOCS, suppressor of cytokine signaling

The relationships of progesterone and methylation levels of *Chr17:76356190* and *Chr17:76356199* with glucose metabolism markers are displayed in Fig. 1. After adjusting multiple confounding factors, progesterone was positively associated with FPG, HbA1c, and Ln-HOMA2-IR, whereas negatively associated with Ln-HOMA2-β in both men and postmenopausal women. On the contrary, *Chr17:76356190* methylation was negatively associated with HbA1c as well as Ln-HOMA2-IR among both men and postmenopausal women, apart from, negatively associated with FPG, and positively associated with Ln-HOMA2-β among postmenopausal women. Meanwhile, *Chr17:76356199* methylation was inversely correlated with FPG in men, as well as Ln-HOMA2-IR in postmenopausal women.

### Combination effects

**Fig. 2:**
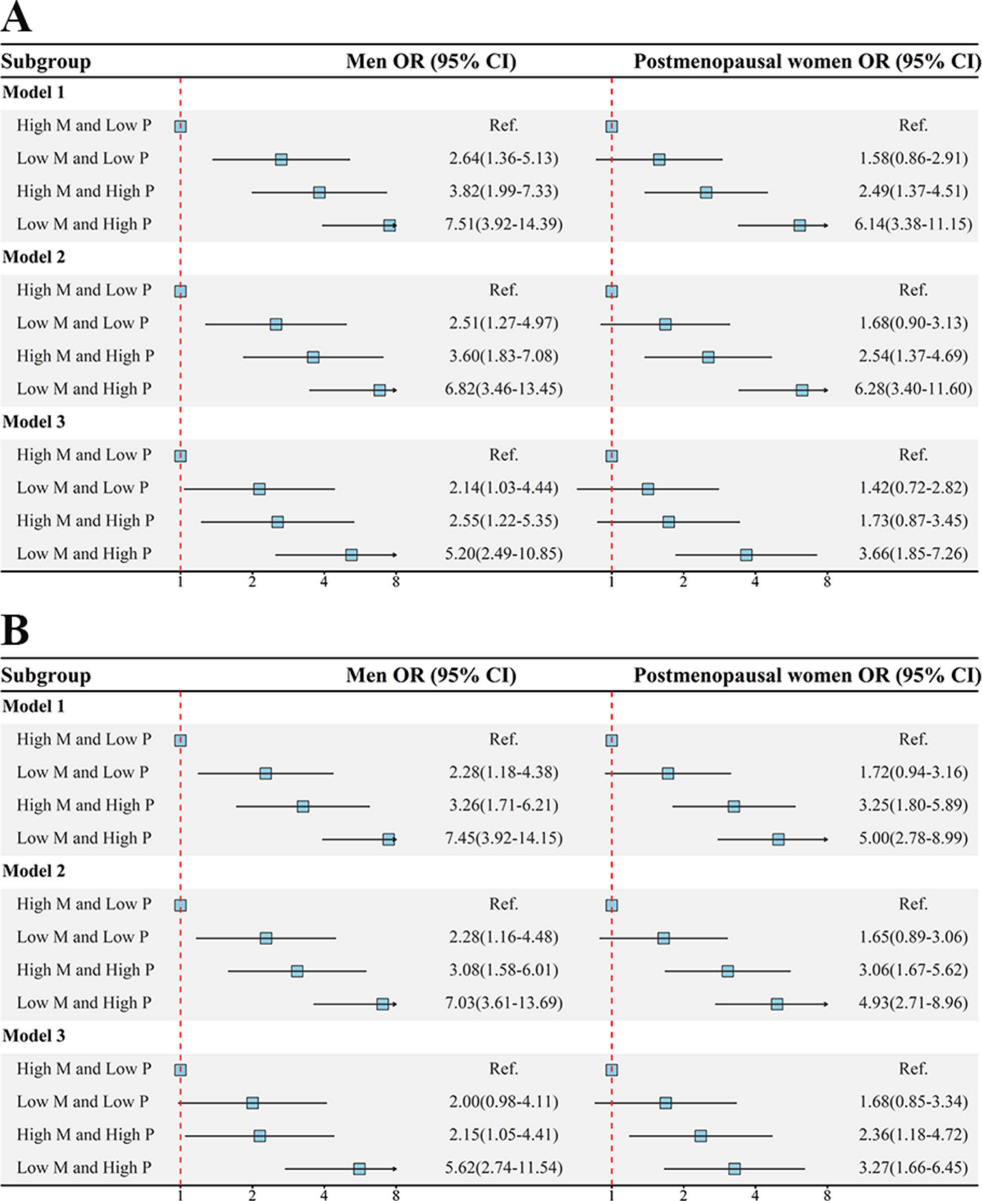
The combined effects of progesterone and SOCS3 methylation (Chr17:76356190 and Chr17:76356199) on T2DM in men and postmenopausal women. “**A**” showed the combined effects of progesterone and Chr17:76356190 methylation on T2DM in men and postmenopausal women; “**B**” showed the combined effects of progesterone and Chr17:76356199 methylation on T2DM in men and postmenopausal women. The arrow indicates that the value in this direction is greater than the length of the line segment. Model 1: no adjust; Model 2: adjusted for age, smoking status, drinking status, physical activity, and family history of T2DM; Model 3: Model 2 + BMI, PP, TC, and TG. CI, confidence interval; OR, odds ratio; M, SOCS3 methylation; P, progesterone

The results of the combined effects of progesterone and methylation level of *Chr17:76356190* or *Chr17:76356199* on T2DM are shown in Fig. 2. After adjusting for confounding factors, the combination effects of progesterone and *Chr17:76356190* or *Chr17:76356199* methylation on T2DM were observed among both men and postmenopausal women, suggesting that individuals who exposed to high levels of progesterone and low level of *Chr17:76356190* or *Chr17:76356199* methylation had a greater risk of prevalence of T2DM than those who exposed to low levels of serum progesterone and high level of *Chr17:76356190* or *Chr17:76356199* methylation (men: OR (95% CI): 5.20 (2.49, 10.85) or 5.62 (2.74, 11.54); postmenopausal women: OR (95% CI): 3.66 (1.85, 7.26) or 3.27 (1.66, 6.45)).

### Interaction effects

As shown in Table 3, there was a strong or slightly significant interaction between progesterone and *Chr17:76356199* or *Chr17: 76356190* methylation on T2DM in postmenopausal women, respectively (*P*-interaction = 0.029, and *P*-interaction = 0.129). Meanwhile, there were an apparent interaction trend in men.

**Table 3:** The multiplication interactive associations of progesterone and methylation level of SOCS3 (Chr17:76356190 or Chr17:76356199) on T2DM

Variables	ORs (95% CIs)		*P***-**interaction
	Progesterone	M_190 or M_199	
*Men*			
Progesterone	2.77 (1.79, 4.29)*		
M_190		0.58 (0.39, 0.86)*	
M_199		0.27 (0.14, 0.51)*	
Progesterone + M_190	2.83 (1.82, 4.40)*	0.55 (0.36, 0.84)*	
Progesterone + M_199	3.00 (1.91, 4.71)*	0.24 (0.12, 0.46)*	
Progesterone + M_190 + Progesterone × M_190	1.98 (0.82, 4.79)*	0.37 (0.14, 0.99)*	0.367
Progesterone + M_199 + Progesterone × M_199	2.28 (0.70, 7.44)	0.17 (0.04, 0.80)	0.625
*Postmenopausal women*			
Progesterone	1.85 (1.26, 2.72)*		
M_190		0.43 (0.29, 0.65)*	
M_199		0.53 (0.28, 0.99)*	
Progesterone + M_190	1.87 (1.27, 2.77)*	0.41 (0.26, 0.62)*	
Progesterone + M_199	1.90 (1.29, 2.81)*	0.47 (0.25, 0.91)*	
Progesterone + M_190 + Progesterone × M_190	3.46 (1.41, 8.51)*	0.74 (0.31, 1.77)	0.129
Progesterone + M_199 + Progesterone × M_199	6.35 (1.95, 20.67)*	1.85 (0.47, 7.24)	0.029

Adjusted for age, smoking status, drinking status, physical activity, family history of T2DM, BMI, PP, TC, and TG

CI, confidence interval; OR, odds ratio; M_190, methylation of Chr17:76,356,190; M_199, methylation of Chr17:76,356,199. *P < 0.05

### Sensitivity analyses

As shown in Additional file 1: Tables S1 and S2, the independent and combined effects of progesterone and *Chr17:76356190* or *Chr17:76356199* methylation on T2DM did not change after excluding postmenopausal women who had taken hormone replacement therapy.

The results of sensitivity analyses by excluding patients with anti-diabetic medication for two weeks are summarized in Additional file 1: Tables S3 and S4. As Additional file 1: Table S3 shows, the associations of progesterone and *Chr17:76356190* methylation with T2DM were remained in men and postmenopausal women, after excluding patients who had taken anti-diabetic medication for two weeks. However, the negative correlation between *Chr17:76356199* methylation and T2DM was only observed in men. As Additional file 1: Table S4 displays, progesterone was positively correlated with FPG, HbA1c, and negatively associated with Ln-HOMA2-β in both men and postmenopausal women. Additionally, positive association between progesterone and Ln-HOMA2-IR was also found among postmenopausal women. *Chr17: 76356190* methylation was negatively correlated with FPG, Ln-INS, and Ln-HOMA2-IR in men; negatively correlated with FPG, HbA1c, and Ln-HOMA2-IR, and positively associated with Ln-HOMA2-β in postmenopausal women. The inverse relationship of *Chr17: 76356199* methylation with FPG was also observed in men.

## Discussion

In the present study, we investigated the independent and combined effects of progesterone and *SOCS3* methylation on T2DM among men and postmenopausal women in Henan rural areas, and discovered that progesterone was associated with an increased risk of T2DM; nevertheless, methylation of *Chr17:76356190* or *Chr17:76356199* was associated with a decreased risk of T2DM. Furthermore, the combined effects of progesterone and *Chr17:76356190* or *Chr17:76356199* methylation on T2DM were observed. Men and postmenopausal women who exposed to high levels of progesterone and low level of *Chr17:76356190* or *Chr17:76356199* methylation had a greater risk of T2DM, compared with those who exposed to low levels of progesterone and high level of *Chr17:76356190* or *Chr17:76356199* methylation.

Research on the relationship between progesterone and diabetes has emerged gradually in recent years. For instance, studies based on animals discovered that increased progesterone levels were associated with hyperglycemic status in female mice [9], and rat treated with progesterone had declined insulin secretion compared to control islets [23]. In the same way, surgical postmenopausal monkeys that treated with medroxyprogesterone acetate and estrogen had decreased insulin sensitivity [24]. Furthermore, a population-based cohort study found that oral contraceptive might increase the risk of diabetes among women aged 40–70 years in Shanghai [25]. Similarly, an longitudinal study followed up 8 years in elderly opposite-sex twins reported that the highest quartile of the serum progesterone distribution was correlated with an increased prevalence of diabetes [26]. Consistent with these results, this research also found the positive association between progesterone and T2DM. Thus, our study supported the existence of relationship between progesterone and T2DM among man and postmenopausal women. An explanation for progesterone effect on T2DM is that progesterone affects the apoptosis and function of pancreatic β-cells by augmenting the generation of oxidative species or increasing the activities of caspases 9, 12 and 3 in RINm5F insulin-producing cells [7]. Moreover, it has been reported that the activation of PI3K and subsequent activation of Akt through the insulin receptor substrate (IRS) signaling pathway are critical for insulin-stimulated GLUT4 translocation [27, 28], and Cb1/TC10 pathway also affects GLUT4 translocation that is insulin-induced [29]. In addition, progesterone can suppress the PI3K pathway by promoting the degradation of IRS-1 and suppress the subsequent Akt phosphorylation, and inhibit GLUT4 translocation and glucose uptake in a step distal to Akt phosphorylation [20]. Thus, progesterone could affect the apoptosis and function of pancreatic β-cells or lead to the development of insulin resistance, consequently T2DM. However, the exact biological mechanisms of progesterone affecting the pathogenesis of T2DM are still unclear, and we will further explore it in the following studies.

*SOCS3* methylation was found to have a significantly negative association with prevalence of T2DM in this present study. Similar results were reported in previous studies [22, 30–34]. For example, available evidence from a nested case–control study in Asians and Europeans suggested that *SOCS3* methylation was associated with future T2DM, and the relative risk was 0.94 (0.92–0.96) [30]. A recent study has demonstrated that increased *SOCS3* expression was correlated with IR in obese individuals and attenuation of *SOCS3* expression could improve glucose tolerance and insulin sensitivity [31]. The same results were also observed in animal trials [32]. Furthermore, therapies designed to suppress *SOCS3* in skeletal muscle might be effective in reversing obesity-related glucose intolerance and IR [33]. Moreover, as known, methylation could silence genes and thus suppress their expression [35]. Thereby, these results indicated that *SOCS3* methylation might alleviate IR by reducing SOCS3 protein expression, which supported the findings of this study to a certain extent. As for the responsible mechanisms, it has been proposed that overexpression of *SOCS3* decreased IRS-1 and IRS-2 tyrosine phosphorylation [36], promoted IRS protein degradation [37, 38], and inhibited PI3K/Akt pathway [18, 19], thus leading to IR and consequently causing T2DM.

**Fig. 3:**
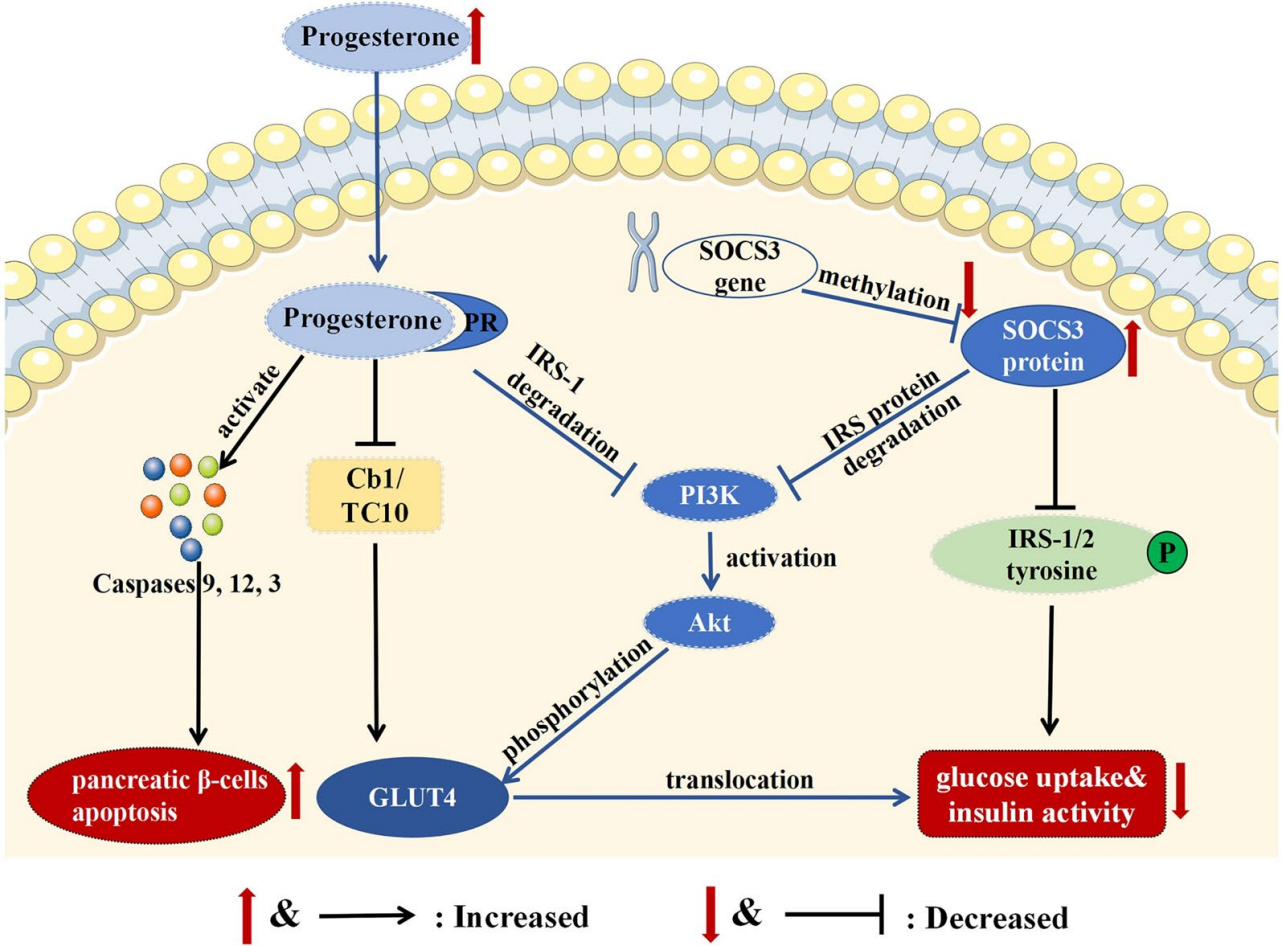
The potential biological mechanisms of progesterone and DNA methylation of SOCS3 gene on T2DM. GLUT4: glucose transporter type 4; IR: insulin resistance; IRS: insulin receptor substrate; PR: progesterone receptor; PI3K: phosphatidylinositol 3-kinase; SOCS3, suppressor of cytokine signaling 3

Interestingly, this study found the combined effects of progesterone and *SOCS3* methylation on T2DM among both men and postmenopausal women. Compared to individuals with high level of *Chr17:76356190* or *Chr17:76356199* (*SOCS3*) methylation and low levels of progesterone, those with low level of *Chr17:76356190* or *Chr17:76356199* (*SOCS3*) methylation and high levels of progesterone had a greater risk of prevalent T2DM. Taken together, a reasonable explanation for the combined effect of progesterone and *SOCS3* methylation on T2DM was that low methylation of *SOCS3* gene leads to high expression of its protein, and SOCS3 protein and progesterone result in IR though suppressing the PI3K/Akt pathway, as Fig. 3 displays. However, the specific biological mechanism of the combined effects of progesterone and *SOCS3* methylation on T2DM remains unclear, and prospective studies with more rigorous design are needed.

The associations of progesterone or *SOCS3* methylation with T2DM and glucose metabolism markers were not completely consistent. This inconsistency might be due to that the participants with T2DM in this study were not all new cases, and some of the T2DM patients were taking one or more than one medication or changing their lifestyles to control hyperglycemia, and maintain glucose metabolism markers at relatively normal levels; thus, the levels of glucose metabolism marker at the time of detection cannot accurately reflect the levels at the onset of the disease. Although the sensitivity analysis was conducted by excluding patients with anti-diabetic medication for two weeks, it only controlled for the effect of taking hypoglycemic drugs to a certain extent, and the effect of lifestyle changes or other unadjusted confounding factors on the results could not rule out. Therefore, follow-up cohort studies are necessary to clearly explore the associations of progesterone and *SOCS3* methylation levels with glucose metabolism indicators.

This study had some strengths: First of all, serum progesterone levels were determined with LC–MS/MS, which minimized the potential for measurement bias. Moreover, to our known, this study was the first one to explore the combined effects of progesterone and *SOCS3* methylation on T2DM to data. Nevertheless, we acknowledge the following limitations of this study. Firstly, it was case–control rather longitudinal design, and thus reverse causality cannot be excluded. Secondly, the *SOCS3* gene methylation was detected with whole blood, and it might vary between different leucocyte subtypes. Thirdly, we only evaluated a subset of factors that were known to be related to T2DM, and could not rule out the influence of other factors. Fourthly, although we controlled several factors related to high level of progesterone, there still exist unknown factors which are not in our consideration influencing the relationship of progesterone with T2DM. Finally, the population of this study were from rural areas of China; thus, it was limited to extend to urban areas and other ethnic groups.

## Conclusions

In conclusion, the high levels of serum progesterone and low level of *Chr17:76356199* or *Chr17:76356199* (*SOCS3*) methylation might contribute to the increased risk of T2DM in Chinese rural population. Moreover, compared with those who exposed to low levels of serum progesterone and high level of *Chr17:76356190* or *Chr17:76356199* (*SOCS3*) methylation, individuals who exposed to high levels of progesterone and low level of *Chr17:76356190* or *Chr17:76356199* (*SOCS3*) methylation had the highest risk of prevalent T2DM. Collectively, ensuring low levels of progesterone and high methylation of *SOCS3* could reduce the prevalence of T2DM. More importantly, this study laid the foundation for the study of the mechanisms of progesterone and *SOCS3* gene on T2DM; however, further multicenter prospective studies are needed to determine the exact underlying mechanisms of diabetes development.

## Methods

### Study population

Participants in this study were drawn from the Henan Rural cohort (Registration Number: ChiCTR-OOC-15006699), which is an ongoing prospective population study, and the details of this study have been reported previously [39]. In brief, this cohort was established in five rural regions (Suiping county, Xinxiang county, Yuzhou county, Tongxu county, and Yima county) of Henan province in China between July 2015 and September 2017, and a multistage, stratified cluster sampling method was applied to obtain samples in the general population. The target population was adults aged 18–79 years who were permanent residents and available for completing follow-up studies of mortality and morbidity. Ultimately, 39,259 adults aged 18–79 years were recruited for the cohort study. In this case–control study, a total of 378 T2DM patients and 679 controls were selected randomly at first, whereas premenopausal women and women without menopausal information (*n* = 109) were excluded, because of the significant effect of the menstrual cycle on progesterone levels, moreover, participants without information of progesterone (*n* = 7) or methylation level of *SOCS3* gene (*n* = 27) were also excluded. Ultimately, a total of 914 men and postmenopausal women were recruited, including 157 men with T2DM, 286 men controls, 172 postmenopausal women with T2DM, and 299 postmenopausal women controls. The study was executed with the approval of the Life Science Ethics Committee of Zhengzhou University (Code: [2015] MEC (S128)), and in accordance with the Helsinki Declaration. Moreover, written informed consent was obtained from all participants.

### Serum progesterone measurement

The venous blood samples from all participants were collected after fasting for more than 8 h at night, and placed at -80℃ before analysis. Liquid chromatography-tandem mass spectrometry (LC–MS/MS) (Waters XEVO TQD system (Waters, Milford, MA, USA)) was used to detect the concentrations of serum progesterone. For the purpose of quality control, 12 samples were tested for each batch; in addition, 1 blank sample and 1 control sample were tested before each batch. Moreover, the value of 1/2 limit of detection (LOD) was used for analysis when the sample serum progesterone level was lower than limit of quantitation (LOQ) [40]. Furthermore, the individuals of the present study were divided into three groups according to the tertile (T) of serum progesterone, and the lowest level (T1) was deemed as reference.

### DNA extraction and methylation analysis of SOCS3

The genomic DNA was evaluated using whole blood of each individual using Whole Blood Genomic DNA Extraction Kit III (Magnetic bead) (Bioteke Corporation, Beijing, China) following the instructions of manufacturer. According to the criteria of length > 200 bp, percent C + percent G > 50.00%, and observed/expected ration > 0.60, two CpG regions were sequenced from CpG islands of *SOCS3* gene. Additionally, they were length 314 (76354776–76355089) and length 1423 (76355093–76356515), respectively. Moreover, the shorter CpG island was designed a pair of primers and the longer CpG inland was designed two pairs of primers for detection. Since previous literature reported that the *Chr17:76354621* site (*cg18181703*) was significantly correlated with T2DM, primer was designed for nearby sequences including this site. Thus, a total of 4 target regions and 93 CpG sites were included in this study.

To detect the methylation of *SOCS3* gene, first of all, genomic DNA was subjected to sodium bisulfite treatment using the EZ DNA Methylation-Gold Kit (ZYMO, CA, USA) to convert the unmethylated C into U in the sample. Then, optimized primers were mixed to form multiplex PCR primer panel, and the multiplex PCR technique was used to amplify genomic DNA. Each PCR reaction mixture was 20 μl, and included 1 × reaction buffer (Takara, Dalian, China), 2.0 mM Mg^2+^, 0.2 mM dNTP, 0.1 μM of each primer, 1 U HotStarTaq polymerase (Takara, Dalian, China), and 2.0 μl template DNA. The cycling program was 95 °C for 2 min; 11 cycles of 94 °C for 20 s, 63 °C for 40 s with a decreasing temperature step of 0.5 °C per cycle, 72 °C for 1 min; then followed by 24 cycles of 94 °C for 20 s, 65 °C for 30 s, 72 °C for 1 min; 72 °C for 2 min. Then, 1 μl diluted PCR amplicons were used for index PCR reaction in 20 μl mixture, including 1 × reaction buffer, 0.3 mM dNTP, 0.25 μM F primer, 0.25 μM index primer, 1 U Q5TM DNA polymerase, and 1.0 μl template DNA. The cycling program was 98 °C for 30 s; 11 cycles of 98 °C for 10 s, 65 °C for 30 s, and 72 °C for 30 s; 72 °C for 5 min. Afterwards, libraries from different samples were quantified and pooled together, followed by sequencing on the Illumina Hiseq (Illumina, San Diego, CA, USA) platform in according with protocols of the manufacturer.

Further, the DNA methylation level of *SOCS3* gene (including 4 target regions and 93 CpG sites) was assessed with Methy1Target™ (Genesky Corporation, Shanghai, China) [22, 34].

### Definition of T2DM

In accordance with the diagnostic criteria of American Diabetes Association (ADA) (2009) [41] and the World Health Organization (WHO) (1999) [42], after excluding type 1 diabetes mellitus and diabetes caused by other reasons, those people who satisfy one of the following criteria were deemed as T2DM patients: (1) FPG ≥ 7.0 mmol/L; (2) HbA1c ≥ 6.5%; (3) having a self‐reported previous diagnosis of diabetes by a physician and taking anti-glycemic agents in the last two weeks.

### Definition of covariates

Information of the participants was collected with a structured questionnaire, including sociodemographic characteristics (age, level of education, and marital status), lifestyles (physical activity, smoking and drinking status, and vegetable and fruit intake), and history of disease. Briefly, the daily threshold for intaking more fruit and vegetable was 500 g, according to the dietary guidelines for Chinese residents. Moreover, participants were considered to have a family history of T2DM if they had a parent with diabetes. For women, menopausal status was asked additionally, and menopausal status was defined as the cessation of menstrual bleeding for more than 12 months. The details have been previously reported [22].

Moreover, anthropometric measurements were taken in this study. In detail, BMI was calculated as the weight (kg) divided by the square of height (m). PP was systemic blood pressure (SBP) minus diastolic blood pressure (DBP). The homeostasis model assessment (HOMA)2-IR and HOMA2-β, which represented the degree of IR and β-cell function, were evaluated by the updated homeostasis model assessment [43]. HbA1c was measured by enzymatic hydrolysis with an automatic analyzer. INS was determined by radioimmunoassay. FPG, TG, and TC were detected with Roche Cobasc501 automatic biochemical analyzer.

### Statistical analysis

The characteristics of the participants were summarized separately within each randomized group by using means and corresponding standard deviations (SDs) (continuous variables, normal distribution), medians and corresponding interquartile ranges (IQRs) (continuous variables, skewed distribution), or numbers and corresponding percentages (categorical variables), respectively. Additionally, t tests, Mann–Whitney *U* tests, and Chi-square tests were employed to compare the differences of normal continuous, skewed continuous, and categorized variables between case and control groups, respectively.

Univariate logistic regression was utilized to investigate the associations of methylation of 4 gene regions and 93 CpG sites of *SOCS3* with T2DM, and a *P* < 0.05/4 and *P* < 0.05/93 in two sides were seen statistically significant. Based on these, two gene sites of *SOCS3* (*Chr17:76356190* and *Chr17:76356199*) were significant correlated with T2DM, and the results had been reported in our previous research [22].

Since the levels of progesterone vary between men and postmenopausal women, logistic regression models were used to examine the effects of progesterone and methylation levels of *Chr17:76356190* and *Chr17:76356199* on T2DM in men and postmenopausal women, respectively. *Odds ratios* (ORs) and 95% confidence intervals (*CI*s) of T2DM were reported. Furthermore, a *P* for trend was calculated in logistic regression models by using the ordinal scoring of the increasing exposure categories without the reference category. Multivariable adjustment models were performed:

Model 1: no adjustment.

Model 2: adjusted for age, smoking status, drinking status, physical activity, and family history of T2DM.

Model 3: Model 2 + BMI, PP, TC, and TG.

Since INS, HOMA2-β, and HOMA2-IR were skewed distribution, natural log (ln-) conversions were performed before the analysis. Then, linear regression was performed to further explore the relationships of progesterone and methylation levels of *Chr17:76356190* and *Chr17:76356199* on markers of glucose metabolism (FPG, HbA1c, Ln-INS, Ln-HOMA2-β, and Ln-HOMA2-IR), after adjusting multiple confounding factors.

Furthermore, progesterone and *SOCS3* methylation were dichotomized into high and low levels based on a median split, to assess the combined effects of progesterone and methylation level of *Chr17:76356190* or *Chr17:76356199* on T2DM with logistic regression in model 3. Additionally, generalized linear model was utilized to estimate the interactive effects of progesterone and *Chr17:76356190* or *Chr17:76356199* methylation on T2DM.

Considering that hormone replacement therapy might affect serum progesterone levels among postmenopausal women, sensitivity analyses were conducted by excluding participants taking hormone replacement to assess the robustness of the effect estimations. Likewise, since T2DM patients were taking one or more than one medication to control hyperglycemia, anti-diabetic medication might have effect on markers of glucose metabolism. After excluding patients with anti-diabetic medication for two weeks, we repeated analyses of the associations of progesterone and *Chr17:76356190* and *Chr17:76356199* methylation with T2DM as well as glucose metabolism markers with logistic regression and linear regression models, respectively.

All statistical analyses were carried out with SPSS (version 21.0) and R software (version 3.6.2), and the significance level was set at *P*<0.05 (two-tailed).

## Supplementary Information

**Additional file 1**. **Table S1**. Associations of progesterone and methylation level of SOCS3 with T2DM in postmenopausal women without taking hormone replacement therapy. **Table S2**. The combined effects of progesterone and SOCS3 methylation (Chr17:76356190 and Chr17:76356199) on T2DM in postmenopausal women without taking hormone replacement therapy. **Table S3**. Associations of progesterone and methylation levels of Chr17:76356190 and Chr17:76356199 with T2DM in participants without taking anti-diabetic medication. **Table S4**. The β cofficients (95% CIs) in markers of glucose metabolism associated with serum progesterone concentrations and methylation level of SOCS3 (Chr17:76356190 and Chr17:76356199) in participants without taking anti-diabetic medication.

## Data Availability

The datasets used and/or analyzed during the current study are available from the corresponding author on reasonable request.

## Abbreviations

SOCS: Suppressor of cytokine signaling
T2DM: Type 2 diabetes mellitus
IR: Insulin resistance
OR: Odds ratio
CI: Confidence interval
CIS: Cytokine-inducible SH2-containing protein
QUICKI: Quantitative insulin sensitivity check index
PI3K: Phosphatidylinositol 3-kinase
LC–MS/MS: Liquid chromatography-tandem mass spectrometry
Ln-: Natural log
LOD: Limit of detection
LOQ: Limit of quantitation
T: Tertiles
BMI: Body mass index
PP: Pulse pressure
DBP: Diastolic blood pressure
SBP: Systemic blood pressure
HOMA: Homeostasis model assessment
HbA1c: Glycosylated hemoglobin A1c
INS: Insulin
FPG: Fasting plasma glucose
TC: Total cholesterol
TG: Triglyceride
SD: Standard deviations
IQR: Interquartile ranges
GLUT: Glucose transporter type
IRS: Insulin receptor substrate
