# Supplementary material for: Combined effects of progesterone and SOCS3 DNA methylation on T2DM: a case–control study
Source: Clin Epigenetics. 2021 Sep 26;13:181. doi: 10.1186/s13148-021-01172-9 (PMC8474856; doi:10.1186/s13148-021-01172-9)
Supplement: Supplementary file 1 — Additional file 1. Table S1. Associations of progesterone and methylation level of SOCS3 with T2DM in postmenopausal women without taking hormone replacement therapy. Table S2. The combined effects of progesterone and SOCS3 methylation (Chr17:76356190 and Chr17:76356199) on T2DM in postmenopausal women without taking hormone replacement therapy. Table S3. Associations of progesterone and methylation levels of Chr17:76356190 and Chr17:76356199 with T2DM in participants without taking anti-diabetic medication. Table S4. The β cofficients (95% CIs) in markers of glucose metabolism associated with serum progesterone concentrations and methylation level of SOCS3 (Chr17:76356190 and Chr17:76356199) in participants without taking anti-diabetic medication. [file 13148_2021_1172_MOESM1_ESM.docx]

**Legends for supplementary tables**

**Supplementary Table 1** Associations of progesterone and methylation level of SOCS3 with T2DM in postmenopausal women without taking hormone replacement therapy

**Supplementary Table 2** The combined effects of progesterone and SOCS3 methylation (Chr17:76356190 and Chr17:76356199) on T2DM in postmenopausal women without taking hormone replacement therapy

**Supplementary Table 3** Associations of progesterone and methylation levels of Chr17:76356190 and Chr17:76356199 with T2DM in participants without taking anti-diabetic medication

**Supplementary Table 4** The β cofficients (95% CIs) in markers of glucose metabolism associated with serum progesterone concentrations and methylation level of SOCS3(Chr17:76356190 and Chr17:76356199) in participants without taking anti-diabetic medication

**Supplementary Table 1** Associations of progesterone and methylation level of SOCS3 with T2DM in postmenopausal women without taking hormone replacement therapy

| **Groups** | ***OR*s (95%*CI*s)** | | |
| --- | --- | --- | --- |
|  | **Model 1** | **Model 2** | **Model 3** |
| **Progesterone** |  |  |  |
| continuous | 2.37 (1.65, 3.39)* | 2.35 (1.63, 3.39)* | 1.85 (1.26, 2.71)* |
| T1≤0.78 | Reference | Reference | Reference |
| T2 0.78~1.18 | 2.14 (1.28, 3.58)* | 2.23 (1.32, 3.77)* | 1.84 (1.02, 3.32)* |
| T3>1.18 | 4.71 (2.84, 7.81)* | 4.69 (2.79, 7.90)* | 3.21 (1.77, 5.80)* |
| *P*-trend | <0.001 | <0.001 | <0.001 |
| **Methylation of SOCS3(Chr17:76356190)** | |  |  |
| continuous | 0.44 (0.31, 0.64)* | 0.43 (0.29, 0.63)* | 0.44 (0.29, 0.67)* |
| T1≤0.69 | Reference | Reference | Reference |
| T2 0.69~1.08 | 1.05 (0.67, 1.66) | 1.05 (0.66, 1.67) | 1.21 (0.71, 2.04) |
| T3>1.08 | 0.46 (0.28, 0.75)* | 0.45 (0.27, 0.75)* | 0.49 (0.28, 0.87)* |
| *P*-trend | 0.002 | 0.003 | 0.020 |
| **Methylation of SOCS3(Chr17:76356199)** | |  |  |
| continuous | 0.47 (0.27, 0.82)* | 0.47 (0.26, 0.83)* | 0.54 (0.29, 1.02) |
| T1≤0.75 | Reference | Reference | Reference |
| T2 0.75~1.04 | 1.03 (0.66, 1.62) | 1.08 (0.68, 1.72) | 1.11 (0.66, 1.88) |
| T2 >1.04 | 0.54 (0.33, 0.87)* | 0.51 (0.31, 0.83)* | 0.61 (0.35, 1.05) |
| *P*-trend | 0.013 | 0.009 | 0.092 |
| Model 1: no adjusted | | | |
| Model 2: Model 1 + age, smoking status, drinking status, physical activity, family history of T2DM | | | |
| Model 3: Model 2 + BMI, PP, TC, TG | | | |

**Supplementary Table 2** The combined effects of progesterone and SOCS3 methylation (Chr17:76356190 and Chr17:76356199) on T2DM in postmenopausal women without taking hormone replacement therapy

| **Model** | **Subgroups** | ***ORs (95%CIs)*** | ***P*** |
| --- | --- | --- | --- |
| ***Methylation of Chr17:76356190 & Progesterone*** | | | |
| **Model 1** | High M and Low P | Reference |  |
|  | Low M and Low P | 1.66 (0.89, 3.08) | 0.110 |
|  | High M and High P | 2.61 (1.43, 4.75)* | 0.002 |
|  | Low M and High P | 6.19 (3.36, 11.40)* | <0.001 |
| **Model 2** | High M and Low P | Reference |  |
|  | Low M and Low P | 1.77 (0.94, 3.33) | 0.079 |
|  | High M and High P | 2.68 (1.44, 5.00)* | 0.002 |
|  | Low M and High P | 6.38 (3.40, 11.99)* | <0.001 |
| **Model 3** | High M and Low P | Reference |  |
|  | Low M and Low P | 1.48 (0.74, 2.96) | 0.267 |
|  | High M and High P | 1.82 (0.91, 3.63) | 0.091 |
|  | Low M and High P | 3.77 (1.88, 7.56)* | <0.001 |
| ***Methylation of Chr17:76356199 & Progesterone*** | | | |
| **Model 1** | High M and Low P | Reference |  |
|  | Low M and Low P | 1.81 (0.97, 3.35) | 0.061 |
|  | High M and High P | 3.33 (1.83, 6.08)* | <0.001 |
|  | Low M and High P | 5.07 (2.78, 9.23)* | <0.001 |
| **Model 2** | High M and Low P | Reference |  |
|  | Low M and Low P | 1.73 (0.92, 3.24) | 0.087 |
|  | High M and High P | 3.17 (1.71, 5.86)* | <0.001 |
|  | Low M and High P | 5.01 (2.72, 9.22)* | <0.001 |
| **Model 3** | High M and Low P | Reference |  |
|  | Low M and Low P | 1.75 (0.88, 3.50) | 0.114 |
|  | High M and High P | 2.48 (1.24, 4.97)* | 0.011 |
|  | Low M and High P | 3.32 (1.67, 6.62)* | 0.001 |
| Model 1: no adjusted | | | |
| Model 2: Model 1 + age, smoking status, drinking status, physical activity, family history of T2DM | | | |
| Model 3: Model 2 + BMI, PP, TC, TG | | | |
| Abbreviations: CI, confidence interval; OR, odds ratio; M, SOCS3 methylation; P, progesterone. | | | |

**Supplementary Table 3** Associations of progesterone and methylation levels of Chr17:76356190 and Chr17:76356199 with T2DM in participants without taking anti-diabetic medication

| **Groups** | **Men** | **Postmenopausal women** |
| --- | --- | --- |
| **Progesterone** |  |  |
| continuous | 2.67 (1.56, 4.57)* | 1.64 (1.09, 2.47)* |
| T1 | Reference | Reference |
| T2 | 1.21 (0.52, 2.80) | 0.98 (0.42, 2.31) |
| T3 | 3.59 (1.62, 7.92)* | 2.81 (1.28, 6.19)* |
| *P*-trend | 0.001 | 0.004 |
| **Methylation of SOCS3(Chr17:76356190)** | |  |
| continuous | 0.55 (0.32, 0.94)* | 0.39 (0.22, 0.67)* |
| T1 | Reference | Reference |
| T2 | 0.98 (0.50, 1.91) | 1.28 (0.64, 2.55) |
| T3 | 0.35 (0.16, 0.77)* | 0.44 (0.20, 0.98)* |
| *P*-trend | 0.012 | 0.069 |
| **Methylation of SOCS3(Chr17:76356199)** | |  |
| continuous | 0.23 (0.10, 0.53)* | 0.54 (0.24, 1.20) |
| T1 | Reference | Reference |
| T2 | 0.51 (0.26, 1.02) | 1.11 (0.55, 2.24) |
| T3 | 0.32 (0.15, 0.67)* | 0.69 (0.32, 1.46) |
| *P*-trend | 0.002 | 0.360 |

**P* < 0.05

Abbreviation: CIs, confidence intervals; ORs, odds ratios; SOCS3, suppressor of cytokine signaling 3; T2DM, type 2 diabetes mellitus; T: tertiles.

**Supplementary Table 4** The β cofficients (95% CIs) in markers of glucose metabolism associated with serum progesterone concentrations and methylation level of SOCS3(Chr17:76356190 and Chr17:76356199) in participants without taking anti-diabetic medication

| **Outcomes** | **Progesterone** | |  | **Methylation level of Chr17:76356190** | |  | **Methylation level of Chr17:76356199** | |
| --- | --- | --- | --- | --- | --- | --- | --- | --- |
|  | **Men (N=370)** | **Postmenopausal women (N=379)** |  | **Men (N=369)** | **Postmenopausal women (N=378)** |  | **Men (N=370)** | **Postmenopausal women (N=379)** |
| FPG |  |  |  |  |  |  |  |  |
| Continuous | 0.694 (0.362, 1.025)* | 0.456 (0.191, 0.720)* |  | -0.300 (-0.596, -0.004)* | -0.558 (-0.837, -0.280)* |  | -0.598 (-1.048, -0.148)* | -0.269 (-0.774, 0.236) |
| T1 | Reference | Reference |  | Reference | Reference |  | Reference | Reference |
| T2 | 0.149 (-0.292, 0.590) | 0.057 (-0.408, 0.521) |  | -0.209 (-0.659, 0.240) | -0.211 (-0.670, 0.248) |  | -0.510 (-0.955, -0.064)* | 0.135 (-0.328, 0.599) |
| T3 | 1.013 (0.534, 1.491)* | 0.862 (0.388, 1.336)* |  | -0.489 (-0.937, -0.040)* | -0.760 (-1.221, -0.298)* |  | -0.569 (-1.014, -0.124)* | -0.155 (-0.620, 0.311) |
| HbA1c |  |  |  |  |  |  |  |  |
| Continuous | 0.497 (0.239, 0.755)* | 0.279 (0.083, 0.474)* |  | 0.038 (-0.193, 0.268) | -0.213 (-0.421, -0.005)* |  | -0.095 (-0.446, 0.257) | 0.083 (-0.289, 0.455) |
| T1 | Reference | Reference |  | Reference | Reference |  | Reference | Reference |
| T2 | 0.246 (-0.094, 0.586) | 0.028 (-0.315, 0.370) |  | -0.309 (-0.657, 0.040) | -0.123 (-0.465, 0.220) |  | 0.010 (-0.339, 0.359) | 0.029 (-0.312, 0.371) |
| T3 | 0.884 (0.515, 1.253)* | 0.600 (0.250, 0.950)* |  | -0.035 (-0.384, 0.313) | -0.191 (-0.536, 0.154) |  | -0.080 (-0.429, 0.268) | 0.093 (-0.251, 0.436) |
| Ln-INS |  |  |  |  |  |  |  |  |
| Continuous | 0.041 (-0.042, 0.123) | 0.051 (-0.005, 0.107) |  | -0.083 (-0.155, -0.012)* | -0.044 (-0.104, 0.015) |  | -0.026 (-0.136, 0.085) | -0.087 (-0.193, 0.018) |
| T1 | Reference | Reference |  | Reference | Reference |  | Reference | Reference |
| T2 | -0.044 (-0.154, 0.066) | -0.083 (-0.182, 0.016) |  | -0.029 (-0.138, 0.080) | 0.030 (-0.067, 0.128) |  | -0.028 (-0.138, 0.082) | -0.062 (-0.159, 0.035) |
| T3 | -0.029 (-0.149, 0.090) | 0.009 (-0.092, 0.110) |  | -0.118 (-0.227, -0.009)* | -0.063 (-0.161, 0.035) |  | -0.048 (-0.157, 0.062) | -0.059 (-0.157, 0.038) |
| Ln-HOMA2-β |  |  |  |  |  |  |  |  |
| Continuous | -0.176 (-0.273, -0.079)* | -0.088 (-0.155, -0.021)* |  | 0.023 (-0.063, 0.109) | 0.109 (0.038, 0.180)* |  | 0.105 (-0.027, 0.236) | 0.015 (-0.113, 0.142) |
| T1 | Reference | Reference |  | Reference | Reference |  | Reference | Reference |
| T2 | -0.096 (-0.224, 0.031) | -0.083 (-0.201, 0.035) |  | 0.005 (-0.126, 0.137) | 0.061 (-0.056, 0.178) |  | 0.105 (-0.026, 0.235) | -0.088 (-0.205, 0.028) |
| T3 | -0.312 (-0.451, -0.173)* | -0.214 (-0.335, -0.094)* |  | 0.050 (-0.081, 0.181) | 0.137 (0.019, 0.254)* |  | 0.082 (-0.049, 0.212) | -0.001 (-0.119, 0.116) |
| Ln-HOMA2-IR |  |  |  |  |  |  |  |  |
| Continuous | 0.066 (-0.016, 0.148) | 0.066 (0.010, 0.123)* |  | -0.091 (-0.162, -0.020)* | -0.063 (-0.123, -0.003)* |  | -0.045 (-0.155, 0.065) | -0.103 (-0.210, 0.004) |
| T1 | Reference | Reference |  | Reference | Reference |  | Reference | Reference |
| T2 | -0.038 (-0.147, 0.072) | -0.077 (-0.177, 0.023) |  | -0.037 (-0.145, 0.072) | 0.016 (-0.083, 0.114) |  | -0.048 (-0.157, 0.062) | -0.064 (-0.163, 0.034) |
| T3 | 0.011 (-0.108, 0.130) | 0.044 (-0.058, 0.146) |  | -0.132 (-0.240, -0.023)* | -0.090 (-0.189, 0.009) |  | -0.068 (-0.177, 0.041) | -0.073 (-0.172, 0.026) |

Adjusted age, smoking status, drinking status, physical activity, family history of T2DM, BMI, PP, TC, TG

Abbreviations: CI, confidence interval; FPG, fasting plasma glucose; HbA1c, glycosylated hemoglobin A1c; Ln-: natural log; HOMA: homeostasis model assessment; INS, insulin; IR: insulin resistance; SOCS, suppressor of cytokine signaling.
